# Rheological and Mechanical Properties and Spinning Behavior of a Starch-Based Biodegradable Polymer †

**DOI:** 10.3390/polym16233306

**Published:** 2024-11-27

**Authors:** Marco Morreale, Marilena Baiamonte, Francesco Paolo La Mantia

**Affiliations:** 1 Department of Engineering and Architecture, Kore University of Enna, Cittadella Universitaria, 94100 Enna, Italy; 2 National Interuniversity Consortium of Materials Science and Technology (INSTM), Via Giusti 9, 50121 Florence, Italy; marilena.baiamonte@unipa.it (M.B.); francescopaolo.lamantia@unipa.it (F.P.L.M.); 3 Department of Engineering, University of Palermo, Viale delle Scienze, 90128 Palermo, Italy

**Keywords:** starch-derived polymer, rheological behavior, tensile behavior, non-isothermal elongational flow, fiber spinning, draw ratio

## Abstract

Over the last few years, the interest in biodegradable polymers has been increasing for several reasons, mainly because of the concerns about environmental protection and the reduction of emissions, especially those related to non-renewable fossil-based resources. Therefore, special attention has increased for the development of environment-friendly polymers such as biodegradable/compostable polymers, especially when they come from renewable resources, since this would help in further reducing energy consumption during their life cycle, as well as the overall environmental impact. Thus, every biopolymer should be accurately investigated in terms of its processability and main technological properties in order to find the most suitable applications. In this work, a starch-derived MaterBi^®^ sample was characterized from the rheological and mechanical point of view, with particular focus on its ability to be processed under non-isothermal elongational flow. The role of processing parameters, such as the temperature and humidity content, was investigated, and a significant influence was found from the processing temperature. Fiber spinning was also performed, finding a good spinnability of the extrudates; in this context, the influence of the draw ratio was investigated as well, with significant effects on the main mechanical properties of the fibers.

## 1. Introduction

It is well known that, over the last few years, the interest from both Academia and Industry in biodegradable polymers has been increasing. This can be attributed to several reasons, including the primary importance that the issues about environmental protection and reduction of emissions (especially those related to non-renewable fossil-based resources) have attained. Thus, special attention has increased for the development of environment-friendly polymers, such as biodegradable/compostable polymers. In this context, it is even more important to obtain such materials from renewable resources in order to reduce energy consumption during the life cycle and further diminish the environmental impact [1,2,3,4,5,6,7,8]. It is known that “bioplastics” can be biodegradable but fossil-based (e.g., polybutylene adipate-terephthalate (PBAT), polycaprolactone (PCL)) or biobased (i.e., not based on fossil sources, but bio-derived in terms of source and synthesis, such as biobased polyethylene (PE), polyethylene terephthalate (PET), etc.). The most attractive category in the bioplastics family is the one that includes biodegradable and bio-based polymers, such as poly-lactic acid (PLA), polyhydroxyalkanoates (PHAs), polybutylene succinate (PBS), and starch blends [5].

However, replacing traditional polymers (coming from non-renewable fossil-based resources) with bioplastics, in particular, both biodegradable and biobased bioplastics, is not an easy task. This is true not only with concern for the obvious difficulties in the synthesis and industrial production but also for the technological challenges they must overcome in terms of final properties and processability. For instance, important industrial operations such as film blowing and spinning require suitable rheological properties, which must be, therefore, accurately assessed prior to actual process design. For instance, it is known from the literature [9,10] that rheological parameters such as the melt strength (MS) and the breaking stretching ratio (BSR) are useful tools to evaluate the suitability of film blowing and fiber spinning. An accurate rheological analysis should, indeed, take into account measurements under different shear rates and flow regimes, i.e., shear and non-isothermal elongational flow [9,11,12]. However, even an accurate rheological analysis under different flow regimes may not be sufficient to provide suitable information, which should, therefore, be coupled with laboratory tests to assess the actual processability under the desired conditions (for instance, fiber spinning) in the perspective of industrial-scale production. Only a few papers provide systematic information about the actual processability of biodegradable polymers [9,11,12,13,14,15,16,17,18,19], with multi-faceted and not univocal results, pointing out that every biodegradable polymer should undergo a thorough investigation (primarily rheological but also mechanical) regarding its actual processability and properties under different processing conditions and for different products (e.g., films, fibers, etc.).

With regard to fiber production, the literature provides information about the effects of orientation on the main properties [20] but much less about biodegradable fibers [21,22]. The actual outcomes are strongly dependent on the specific polymer used since the elastic modulus and the tensile strength typically increase upon enhancing the orientation, while the elongation at break decreases: but this usually applies only to semicrystalline polymers, while amorphous polymers and blends may show more complicated patterns, in some cases, including even a brittle to ductile transition induced by the orientation [23]. As previously mentioned, there is not much information available about fiber production from biodegradable polymers [22] and even less regarding an important commercial family known as MaterBi^®^, which typically contains biodegradable polyesters from renewable production sources and has found relevant commercial applications [24,25].

Actually, MaterBis can be starch-based (i.e., containing thermoplastic starch, TPS) or not. In any case, every MaterBi^®^ grade has a different, proprietary formulation and, therefore, should be accurately investigated in terms of processability and the main technological properties in order to find the most suitable applications.

In this work, therefore, we have extensively characterized, from the rheological and mechanical point of view, a starch-derived MaterBi^®^ sample. This choice was driven by the significant bio-based component of the sample and also because of the significant commercial success this polymer family has been achieving, thanks to the wide range of properties (and, therefore, applications) they can assure. In particular, the effects of processing temperature and humidity content on the viscosity under shear and non-isothermal elongational flow were investigated, as well as the actual spinning behavior of the material and the effect of the orientation on the mechanical properties of the filaments (i.e., upon variating the draw ratio). The importance of melt-spun biodegradable fibers in the current technical-scientific scenarios is significant since it is reported that applications could be found in several fields, such as clothing, technical textiles, agriculture, biomedical applications, environmental remediation, etc., and the main difficulties are obtaining good processability and mechanical properties, as well as suitable biodegradation rates [26].

The results showed that this starch-derived compostable polymer is suitable for processing operations where non-isothermal elongational flow is involved.

## 2. Materials and Methods

### 2.1. Materials

The biodegradable polymer investigated in this work belongs to the MaterBi^®^ family produced by Novamont (Novara, Italy). In more detail, it is an EF05B grade. The composition is proprietary, but according to literature data [27], it is composed of biodegradable polyesters, corn starch, and a plasticizer based on polyols.

### 2.2. Processing

The material was used as received. Only an optional drying pre-treatment, in order to perform a comparison with the behavior in the absence of such pre-treatment, was carried out in a vacuum oven at T = 70 °C overnight. As further described in the following section, fibers were produced by spinning the extrudates from a capillary viscometer at different draw ratios.

### 2.3. Characterization

The above-described materials were subjected to rheological characterization in shear flow at lower and higher shear rates performed on a ThermoScientific (Waltham, MA, USA) Mars III rotational plate–plate rheometer and a CEAST (Pianezza, Italy) Rheologic 1000 capillary viscometer respectively, at two different temperatures, namely 145 and 155 °C. Tests at the rotational rheometer were performed on compression-molded samples (diameter = 25 mm, thickness = 1 mm) prepared using a Carver (Wabash, IN, USA) laboratory press set at 150 °C, compression time 3 min. The tests were conducted with a strain equal to 5%, which was chosen after performing a standard procedure based on strain sweep and time sweep tests in order to determine the limits of the linear viscoelastic regime and the maximum test duration (see Supplementary Material). For all the tests performed in the capillary rheometer, a capillary with a diameter (D) of 1 mm and a length-to-diameter (L/D) ratio equal to 40 was used; due to the high length-to-diameter ratio (i.e., 40), Bagley correction was not applied, whereas the Rabinowitsch correction was applied throughout. Rheological characterization was also performed in non-isothermal elongational flow using the same capillary rheometer. In more detail, important rheological properties such as the “melt strength” (MS) and the breaking stretching ratio (BSR) were measured, as described elsewhere [1,10]. All of the rheological tests were performed on at least four samples, with adequate reproducibility of the results (±7%).

Furthermore, the equipment allowed the preparation of fibers at different draw ratios (DR, i.e., the ratio between the capillary diameter and the final diameter of the fiber) for the following mechanical characterization. The latter was carried out on the spun fibers by using an Instron (Norwood, MA, USA) 3365 universal machine at two different speeds, i.e., 1 mm/min up to the first 2 mm of elongation and then 100 mm/min up to fiber rupture. The reproducibility of the results was adequate (±7%). The results were compared with those obtained on isotropic, unoriented samples (90 × 10 × (≈1) mm^3^) obtained by compression molding (similar procedure to the rheology tests specimens). Mechanical tests were performed on at least seven samples, with good reproducibility (±5%).

The role of the humidity content was investigated by performing water absorption and desorption tests: a sample made up of a predetermined amount of virgin pellets was weighed, and then the weight was measured every hour during a desorption cycle (in vacuum oven at 70 °C) for a total of 24 h, and during an absorption cycle (at 20 °C and 60% relative humidity) on the same sample. Adsorption and desorption were evaluated as weight percent gain or loss, respectively, using the following formulas:

AWG = (M_C_ − M_0_)/M_0_ × 100 (1)

DWL = −(M_C_ − M_0_)/M_0_ × 100 (2)

where AWG is the percent weight gain due to absorption, DWL is the percent weight loss due to desorption, M_C_ is the current measured weight of the sample, M_0_ is the initial weight of that sample. The obtained results were the average of at least four samples, and the reproducibility was acceptable (±8%).

Consequently, some samples (i.e., those processed at 155 °C) were tested, taking into account the role of the humidity content by comparing the results obtained on “humid”, i.e., untreated samples, and “dried”, i.e., treated, samples (where the treatment consisted of a 24 h cycle in vacuum oven at 70 °C).

## 3. Results and Discussion

**Figure 1:**
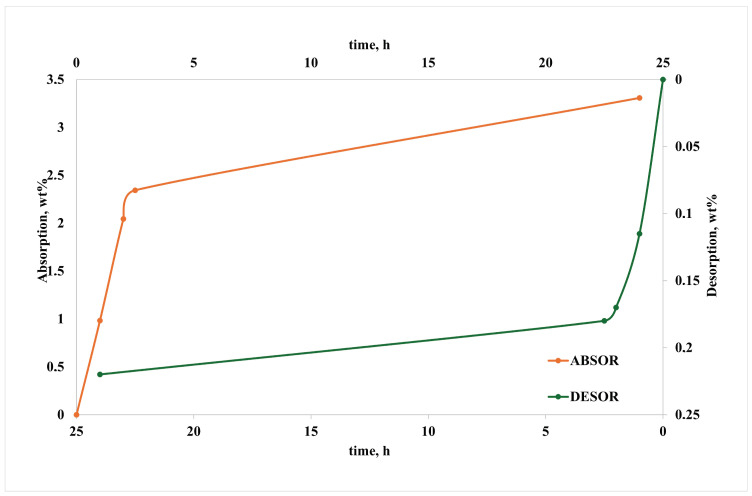
Absorption and desorption kinetic curves.

Figure 1 reports the absorption and desorption curves.

It can be stated that most of the absorption/desorption occurs within a couple of hours and that adsorption/desorption rates, i.e., percent weight variations due to water adsorption or desorption, are relatively low. This suggests that the proprietary formulation (in the above-described terms) succeeds in keeping a suitable hydrophobicity of the polymer system, and therefore, the effect of humidity on the material’s properties is expected to be reasonably restrained.

**Figure 2:**
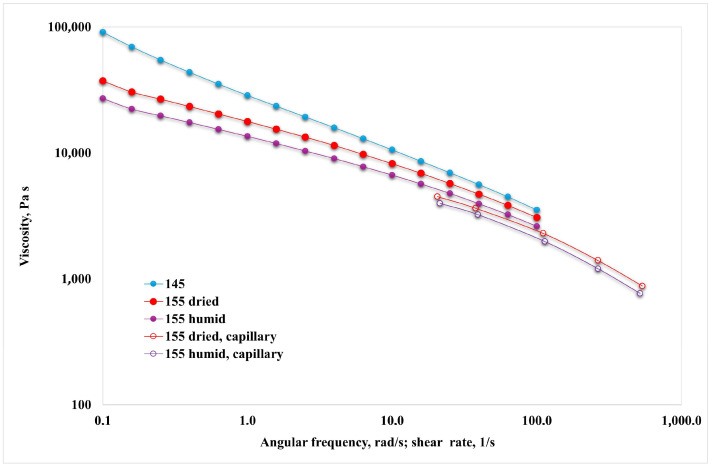
Flow curves of the investigated sample at two temperatures for dried and humid samples. The closed points refer to the data obtained in the rotational rheometer, and the open points to the data obtained in the capillary viscometer.

In Figure 2, the rheological curves at the two temperatures are reported as a function of the frequency and the shear rate for the data obtained in the rotational rheometer and in the capillary viscometer, respectively.

First, it can be observed that all the curves do not attain a Newtonian plateau at low shear rates but, on the other hand, tend to rapidly increase by decreasing the shear rate and show yield stress. This upturn visibly changes when temperature and humidity content change.

Moreover, the two sets of curves (i.e., rotational rheometer vs. capillary viscometer) only approach each other but without superposition. This suggests that the Cox–Merz rule does not apply in this case, as expected based on what was found on relatively similar multiphase heterogeneous systems [1,28]. Regarding the effect of temperature, there are significant differences, with the lower temperature causing not only higher viscosity but also increased yield stress and a stronger non-Newtonian behavior. Concerning the effect of humidity content, the pre-treated (dried) samples showed higher viscosities, increased yield stress, and stronger non-Newtonian behavior. This is certainly linked to both the plasticizing effect of water and likely to some chain scission effects, mainly affecting the polyester component of the polymer.

**Figure 3:**
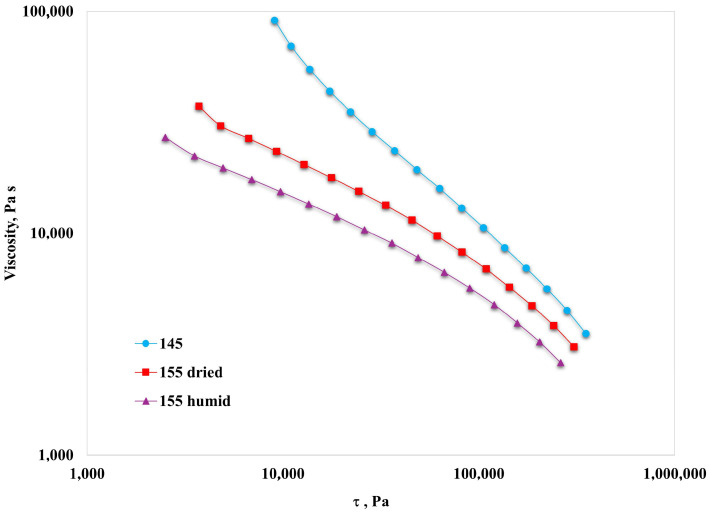
Complex viscosity vs. shear stress.

In Figure 3, the values of the complex viscosity are reported against the shear stress for all the samples.

The above-reported curves suggest the presence of yield stress, where values can be evaluated using the well-known Casson equation, a model used to describe the flow of viscoelastic liquids, in particular with significant yield stress occurrence, and characterized by a more gradual transition from Newtonian behavior to yield [29,30]:

τ^½^ = Y^½^ + (η_PL_γ)^½^ (3)

where Y is the yield stress, η_PL_ is the so-called “Casson plastic viscosity”, and γ is the shear rate [31].

In particular, we calculated the yield stress values at the three main conditions (145 °C, 155 °C on treated, dried samples, and 155 °C on untreated, humid samples), as reported in the following Table 1.

The Y value stands for the minimal stress required to make the polymer system flow. The result found is that, in agreement with the previously reported discussions, yield stress value decreases with the presence of humidity and increasing temperature. This further suggests that the 155 °C temperature should be preferred for actual processing.

**Figure 4:**
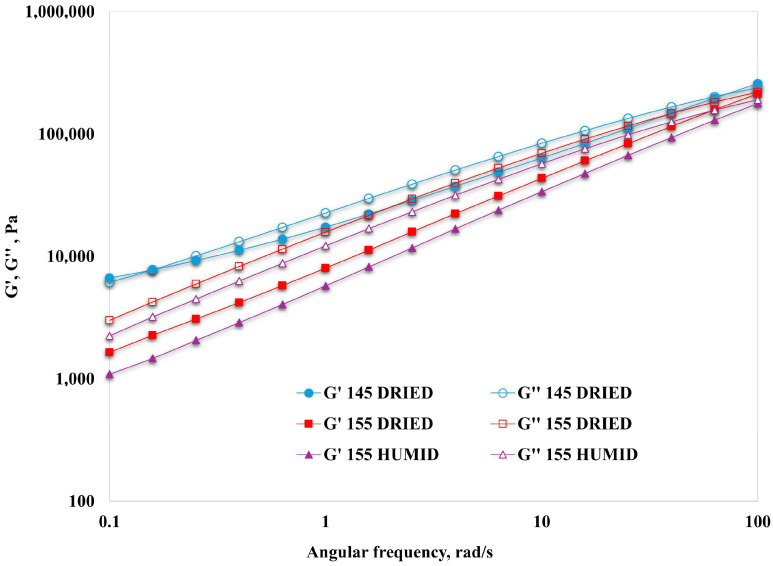
Elastic (G′, full symbols) and storage (G″, empty symbols) moduli of the investigated systems.

The overall trends of G′ and G″ (Figure 4) confirm the previously described behaviors. In more detail, while at higher frequencies, the curves approach each other as expected, and the trends at lower frequencies give more information about the actual behavior and differences, i.e., the drying pre-treatment leads to an increased elastic behavior. However, it should be pointed out that, under the investigated conditions range, the behavior at the higher temperature is liquid-like, with the crossover point occurring around 100 rad/s. In general terms, however, G″ values measured at lower frequencies for each material are higher than G′ values for the same material, suggesting a more liquid-like behavior in that frequency range.

The situation is somewhat different at lower temperatures (i.e., 145 °C), with a significantly more elastic, solid-like behavior at the lower frequencies, confirmed by the relatively higher G′ values and the reduced slopes of the curves [32,33].

**Figure 5:**
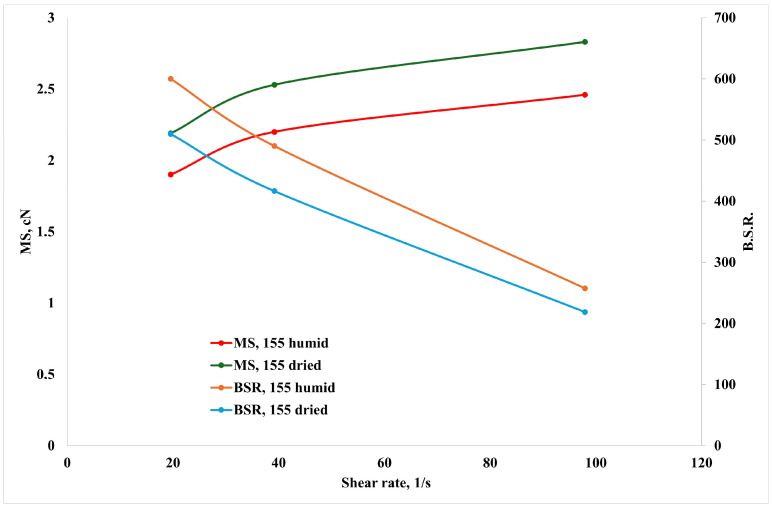
Melt strength (MS) and breaking stretching ratio (BSR) as a function of the shear rate at the temperature of 155 °C.

In Figure 5, the values of the MS and the BSR are reported as a function of the shear rate at the temperature of 155 °C.

The MS and BSR curves show a typical trend, with increasing MS and decreasing BSR upon increasing the shear rate. Although the decreasing trend is remarkable, the absolute BSR values are satisfactory, suggesting good deformability of the melt, and therefore, combined with the good values of the melt strength, the suitability of this material not only for film blowing operations but also for fiber production [9,22].

This was further proved by systematically spinning filaments during the non-isothermal elongational flow tests with different draw ratios, as described in the Section 2. In this case, the sample with a higher BSR, i.e., the humid one, was chosen.

In Table 2, the values of Young’s modulus, E, tensile strength, TS, and elongation at break, EB, of isotropic samples are reported for the dried and humid samples, along with the standard deviations.

It can be observed that the pre-treatment leads, as expected, to an improvement in the tensile properties, although to a very limited extent.

**Figure 6:**
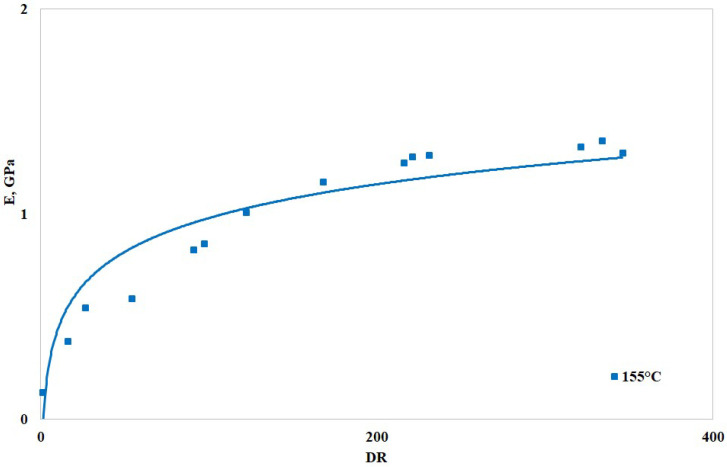
Elastic modulus vs. draw ratio of the spun fibers.

**Figure 7:**
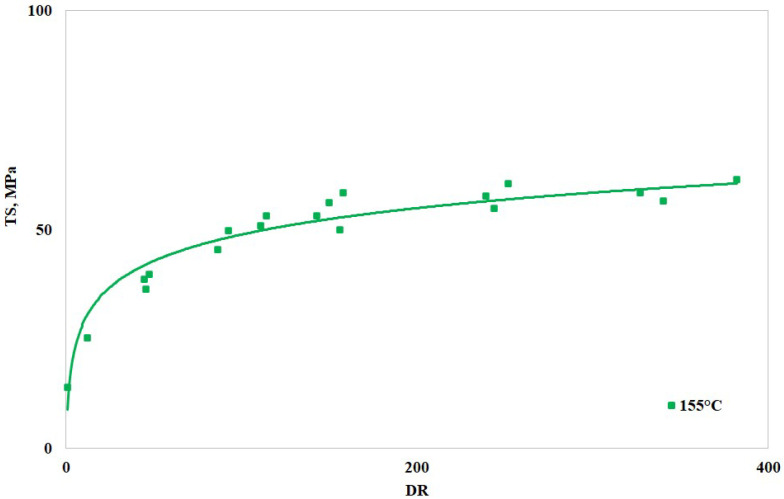
Tensile strength vs. draw ratio of the spun fibers.

**Figure 8:**
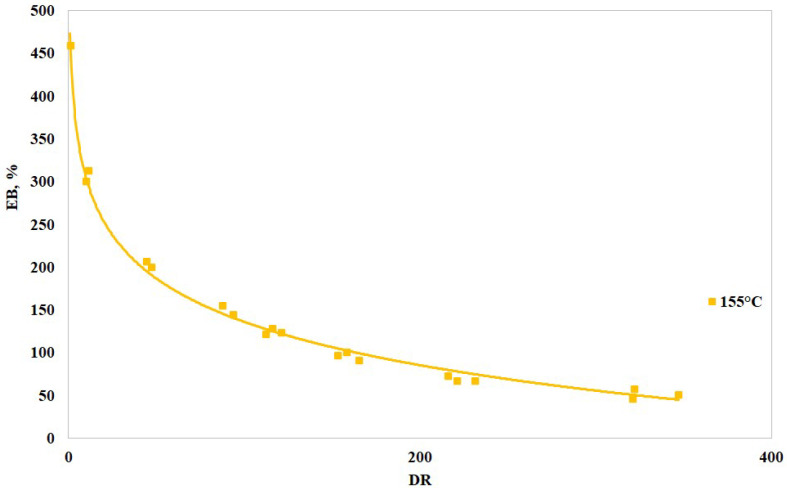
Elongation at break vs. draw ratio of the spun fibers.

In Figure 6, Figure 7 and Figure 8, the values of the elastic modulus, tensile strength, and elongation at break are reported vs. the draw ratio, respectively. More specifically, the figures report the logarithmic interpolation curves (solid lines) of the experimental points (square dots). As anticipated, only the sample with higher BSR (i.e., 155 humid) was tested.

The above-reported curves show that the elastic modulus and the tensile strength increase with the draw ratio while the elongation at break (i.e., the deformability) decreases. Drawing, therefore, increased the orientation degree of the macromolecules and reduced their reciprocal slipping ability. The significant variations found on increasing the DR and the tendency of the curves to reach a plateau (i.e., constant) values suggest that the macromolecules become effectively oriented during the drawing operations, in agreement with the trends found with other similar polymers [1,12]. Most of the improvement is achieved with DR around 90, while higher draw ratios do not increase the values with similar rates. Overall, it can be stated that an increase in the DR effectively improves the mechanical resistance, i.e., elastic modulus and tensile strength, of the spun fibers [34]; the reduction in the elongation at break, which may seem a drawback, is not, since most of the applications involving spun fibers require dimensional stability, and therefore, reasonably low deformability.

## 4. Conclusions

In the present work, a starch-derived MaterBi^®^ was characterized from the rheological and mechanical point of view, with a particular focus on its ability to be processed under non-isothermal elongational flow. In detail, the effects of the processing temperature and humidity content on the viscosity under shear and non-isothermal elongational flow were investigated, as well as the actual behavior under non-isothermal elongational flow of the material and the effect of the orientation on the mechanical properties of the filaments.

The results from the rheological tests showed that a small temperature difference led to a significant increase in the viscosity and a more non-Newtonian behavior, thus suggesting that 155 °C should be the optimal processing temperature. This was further proven under non-isothermal elongational flow since adequate/good values of the melt strength and the breaking stretching ratio were measured. This suggests good processability for film and fiber production. The latter found further confirmation in the very good spinnability of the extrudates.

The obtained filaments were subjected to tensile tests, which showed good values of the elastic modulus and tensile strength, increasing the draw ratio and good deformability, although it was reduced with increasing draw ratio. Potential fields of application of biodegradable melt-spun fibers may include agricultural, biomedical, technical textiles, environmental remediation, biomedical, and clothing.

## Figures and Tables

**Table 1:** Calculated yield stress values of the investigated samples.

Sample	Y [KPa]
145	3.9
155, dried	0.8
155, humid	0.4

**Table 2:** Tensile properties of isotropic samples.

Sample	Elastic Modulus [MPa]	Tensile Strength [MPa]	Elongation at Break [%]
DRIED	128.3 ± 5.1	13.8 ± 0.6	467 ± 16.5
HUMID	125.1 ± 5.9	13.6 ± 0.3	482 ± 13.5

## Data Availability

The original contributions presented in the study are partly included in the article/Supplementary Material, further inquiries can be directed to the corresponding author.

## Supplementary Materials

The following supporting information can be downloaded at: https://www.mdpi.com/article/10.3390/polym16233306/s1, Figure S1: Strain sweep at T = 155 °C; Figure S2: Time sweep at 155 °C.
