# Supplementary material for: Rheological and Mechanical Properties and Spinning Behavior of a Starch-Based Biodegradable Polymer
Source: Polymers (Basel). 2024 Nov 27;16(23):3306. doi: 10.3390/polym16233306 (PMC11644498; doi:10.3390/polym16233306)
Supplement: Supplementary file 1 [file polymers-16-03306-s001.zip › polymers-3262638-supplementary.pdf]

SUPPLEMENTARY DATA

The supplementary data (Figures S1 and S2) show two typical examples of, respectively, the strain and the time sweep tests performed prior to the investigation of main rheological measurements. Fig. S1 shows a strain sweep test performed at 155 °C and 0.1 rad/s frequency on dried samples, while Fig. S2 reports the time sweep test performed at 155 °C, 10 rad/s frequency, 5% strain, on dried samples. The first one suggests that linearity occurs up to approx. 4% strain, then it starts to decrease very slightly; therefore, a 5% strain was considered suitable for the following rheological tests.

The second shows that the obtained viscosity results are steady up to about 200 s, then the viscosity slightly increases, although the effect becomes significant (more than 10% difference) only after approx. 400 s, probably due to crosslinking phenomena.

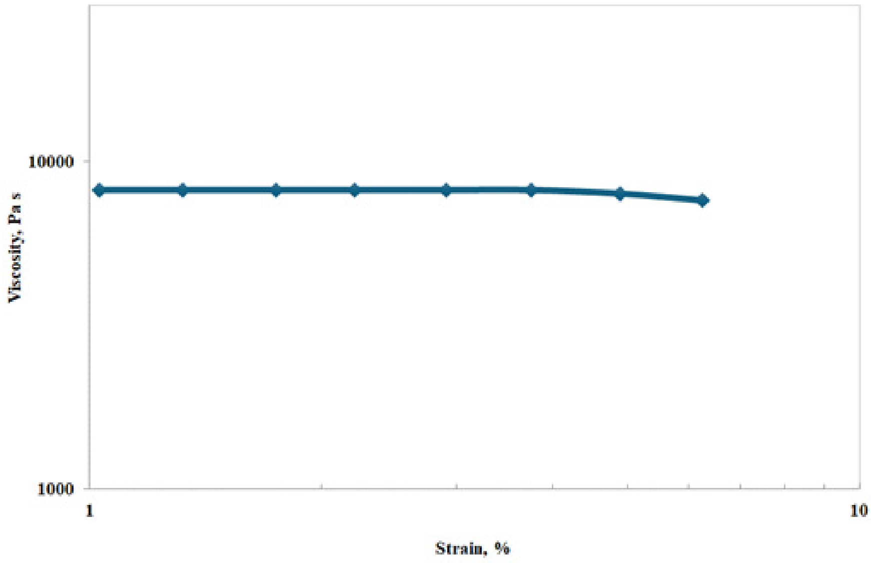

Figure S1. Strain sweep at T = 155 °C.

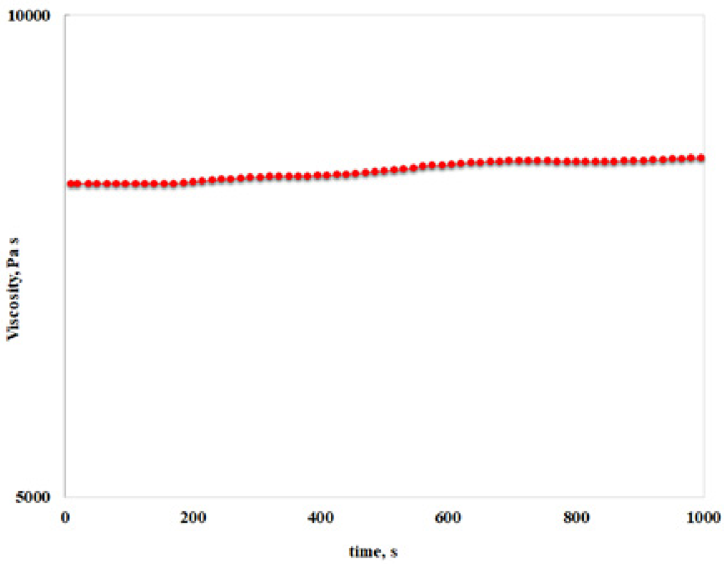

Figure S2. Time sweep at 155 °C
